# *Plasmodium falciparum* susceptibility to standard and potential anti-malarial drugs in Dakar, Senegal, during the 2013–2014 malaria season

**DOI:** 10.1186/s12936-015-0589-3

**Published:** 2015-02-06

**Authors:** Bécaye Fall, Cheikhou Camara, Mansour Fall, Aminata Nakoulima, Pierre Dionne, Bakary Diatta, Yaya Diemé, Boubacar Wade, Bruno Pradines

**Affiliations:** Laboratoire d’étude de la chimiosensibilité du paludisme, Fédération des laboratoires, Hôpital Principal de Dakar, Dakar, Sénégal; Service des Urgences, Hôpital Principal de Dakar, Dakar, Sénégal; Service de Réanimation Médicale, Hôpital Principal de Dakar, Dakar, Sénégal; Service de Pédiatrie, Hôpital Principal de Dakar, Dakar, Sénégal; Maternité Hôpital Principal de Dakar, Dakar, Sénégal; Chefferie, Hôpital Principal de Dakar, Dakar, Sénégal; Unité de Parasitologie et d’Entomologie, Département des Maladies Infectieuses, Institut de Recherche Biomédicale des Armées, Brétigny sur Orge, France; Aix Marseille Université, Unité de Recherche sur les Maladies Infectieuses et Tropicales Emergentes, UM 63, CNRS 7278, IRD 198, Inserm 1095, Marseille, France; Centre National de Référence du Paludisme, Marseille, France

**Keywords:** Malaria, *Plasmodium falciparum*, Anti-malarial, *In vitro*, Resistance, Senegal, Proveblue, Methylene blue

## Abstract

**Background:**

In 2006, the Senegalese National Malaria Control Programme recommended artemisinin-based combination therapy (ACT) as the first-line treatment for uncomplicated malaria. Since the introduction of ACT, there have been very few reports on the level of *Plasmodium falciparum* resistance to anti-malarial drugs. An *ex vivo* susceptibility study was conducted on local isolates obtained from the Hôpital Principal de Dakar (Dakar, Senegal) from November 2013 to January 2014.

**Methods:**

Eighteen *P. falciparum* isolates were sussessfully assessed for *ex vivo* susceptibility to chloroquine (CQ), quinine (QN), monodesethylamodiaquine (MDAQ), the active metabolite of amodiaquine, mefloquine (MQ), lumefantrine (LMF), artesunate (AS), dihydroartemisinin (DHA), the active metabolite of artemisinin derivatives, pyronaridine (PND), piperaquine (PPQ), and, Proveblue (PVB), a methylene blue preparation, using the HRP2-based ELISA test.

**Results:**

The prevalence of isolates with reduced susceptibility was 55.6% for MQ, 50% for CQ, 5.6% for QN and MDAQ, and 0% for DHA, AS and LMF. The mean IC_50_ for PND, PPQ and PVB were 5.8 nM, 32.2 nM and 5.3 nM, respectively.

**Conclusions:**

The prevalence of isolates with a reduced susceptibility to MQ remains high and stable in Dakar. Since 2004, the prevalence of CQ resistance decreased, but rebounded in 2013 in Dakar. PND, PPQ and PVB showed high *in vitro* activity in *P. falciparum* parasites from Dakar.

## Background

In response to increasing chloroquine resistance, Senegal switched in 2004 to sulphadoxine-pyrimethamine with amodiaquine as the first-line therapy for malaria. In 2006, the Senegalese National Malaria Control Programme recommended artemisinin-based combination therapy (ACT) as the first-line treatment for uncomplicated malaria. The combined sulphadoxine-pyrimethamine and amodiaquine treatment was changed to artemether-lumefantrine or artesunate-amodiaquine. Since 2006, more than 1.5 million ACT-based treatments have been administered in Senegal [1]. In 2006, the Senegalese National Malaria Control Programme also recommended testing all suspected cases of malaria with the *Plasmodium falciparum* histidine-rich protein 2 (PfHRP2)-based rapid diagnostic test (RDT). Since this time, ACT use has been restricted to confirmed malaria cases to reduce drug resistance. In 2009, 184,170 doses of ACT were dispensed at public health facilities in Senegal [2].

Since the introduction of ACT, there have been very few reports on the level of *P. falciparum* resistance to anti-malarial drugs. The last *ex vivo* susceptibility study was conducted in 2010 in Dakar (Médina district) [3] and in 2011 in Thies [4]. To determine whether parasite susceptibility has been affected by the new anti-malarial policies, an *ex vivo* susceptibility study was conducted on local isolates from Dakar obtained from the Hôpital Principal de Dakar between November 2013 and January 2014. The malaria isolates were assessed for susceptibility to standard drugs such as chloroquine (CQ), quinine (QN), monodesethylamodiaquine (MDAQ), the active metabolite of amodiaquine, mefloquine (MQ), lumefantrine (LMF), artesunate (AS), dihydroartemisinin (DHA), the active metabolite of artemisinin derivatives, and new anti-malarial drugs, such as pyronaridine (PND), piperaquine (PPQ) and Proveblue (PVB), a methylene blue preparation that complies with the European Pharmacopoeia and contains limited organic impurities and heavy metals of recognised toxicity.

## Methods

### *Plasmodium falciparum* isolates

In total, 24 patients (seven females and 17 males) with malaria were recruited from 19 November 2013 to 7 January 2014 at the Hôpital Principal de Dakar. Venous blood samples were collected in Vacutainer® ACD tubes (Becton Dickinson, Rutherford, NJ, USA) prior to patient treatment. Parasitaemia ranged from 0.001 to 0.33% in the male group and from 0.001 to 3.3% in the female group. Of the 24 patients, 67% were recruited from the emergency department and the remainder were recruited from the intensive care unit (21%), paediatric department (8%) and maternity department (4%). Informed verbal consent was obtained from patients and/or their parents before blood collection. Assessments of *P. falciparum* susceptibility to anti-malarial drugs were performed with the same venous blood sample used for diagnostic purposes. The study was reviewed and approved by the ethical committee of the Hôpital Principal de Dakar. Patients were successfully treated by QN.

Thin blood smears were stained using a RAL® kit (Réactifs RAL, Paris, France) based on eosin and methylene blue and were examined to determine *P. falciparum* density and to confirm monoinfection. Parasitized erythrocytes were washed three times in RPMI 1640 medium (Invitrogen, Paisley, UK) buffered with 25 mM HEPES and 25 mM NaHCO_3_. If parasitaemia exceeded 0.5%, infected erythrocytes were diluted to 0.5% with uninfected erythrocytes (human blood type A+) and resuspended in RPMI 1640 medium supplemented with 10% human serum (Abcys S.A. Paris, France), for a final haematocrit of 1.5%.

### Drugs

CQ, QN and DHA were purchased from Sigma (Saint Louis, MO, USA). MDAQ was obtained from the World Health Organization (Geneva, Switzerland), MQ was purchased from Roche (Paris, France), and LMF was purchased from Novartis Pharma (Basel, Switzerland). AS, PPQ and PND were obtained from Shin Poong Pharm Co (Seoul, Korea) and PVB from Provepharm SAS (Marseille, France). QN, MDAQ, MQ, DHA, AS, and PPQ were first dissolved in methanol and then diluted in water to final concentrations ranging from 6 nM to 3,149 nM for QN, 1.9 to 1,988 nM for MDAQ, 1.5 to 392 nM for MQ, 0.1 to 107 nM for DHA, 0.1 to 98 nM for AS and 1.9 to 998 nM for PPQ. CQ, PND and PVB were resuspended and diluted in water to final concentrations ranging from 6 nM to 3,231 nM, 0.4 to 199 nM and 0.5 to 500 nM, respectively. LMF was resuspended and diluted in ethanol to obtain final concentrations ranging from 0.6 nM to 310 nM.

The batches of plates were tested and validated on the CQ-susceptible 3D7 strain (West-Africa) and the CQ-resistant W2 strain (Indochina) (MR4, Virginia, USA) in three to six independent experiments using the same conditions described in the paragraph below. The two strains were synchronized twice with sorbitol before use [5], and clonality was verified every 15 days using PCR genotyping of the polymorphic genetic markers *msp1* and *msp2* and microsatellite loci [6,7] and annually by an independent laboratory from the Worldwide Anti-malarial Resistance Network (WWARN).

### *Ex vivo* assay

For the *in vitro* isotopic microtests, 100 μl of synchronous parasitized red blood cells (final parasitaemia, 0.5%; final haematocrit, 1.5%) was aliquoted into 96-well plates pre-dosed with anti-malarial drugs. The plates were incubated in a sealed bag for 72 hrs at 37°C with the atmospheric generators for capnophilic bacteria, Genbag CO2® at 5% CO_2_ and 15% O_2_ (BioMérieux, Marcy l’Etoile, France) [8]. After thawing the plates, haemolysed cultures were homogenized by vortexing the plates. Both the success of the drug susceptibility assay and the appropriate volume of haemolysed culture to use for each assay were determined for each clinical isolate during a preliminary HRP2 ELISA. Both the pre-test and subsequent ELISAs were performed using a commercial kit (Malaria Ag Celisa, ref KM2159, Cellabs PTY LTD, Brookvale, Australia) according to the manufacturer’s recommendations. The optical density (OD) of each sample was measured with a spectrophotometer (Multiskan EX, Thermo Scientific, Vantaa, Finland).

The concentration at which the drugs were able to inhibit 50% of parasite growth (IC_50_) was calculated with the inhibitory sigmoid E_max_ model, with estimation of the IC_50_ through non-linear regression using a standard function of the R software (ICEstimator version 1.2) [9]. IC_50_ values were validated only if the OD ratio (OD at concentration 0/OD at concentration max) was greater than 1.6 and the confidence interval ratio (upper 95% confidence interval of the IC_50_ estimation/lower 95% confidence interval of the IC_50_ estimation) was less than 2.0 [9].

### Data and statistical analysis

IC_50_ values were analysed after logarithmic transformation and expressed as the geometric mean of the IC_50_ and a confidence interval of 95% (CI95%).

Using the Genbag conditions, the cut-off values for *in vitro* resistance, or reduced susceptibility, were 77 nM, 61 nM, 115 nM, 12 nM, 12 nM, 611 nM, and 30 nM for CQ, MDAQ, LMF, DHA, AS, QN, and MQ, respectively [10].

## Results

Of the 24 patients recruited at the Hôpital Principal de Dakar, 19 were tested *ex vivo*, and 18 isolates were successfully cultured. The average parameter estimates for the ten anti-malarial drugs used against the *P. falciparum* isolates are given in Table 1. The prevalence of *P. falciparum* isolates with decreased susceptibility to MQ *in vitro* reached 55.6%. Fifty per cent of the isolates were resistant *in vitro* to CQ.

**Table 1:** ***Ex vivo*** **susceptibility of 18** ***Plasmodium falciparum*** **isolates from Dakar to chloroquine (CQ), monodesethylamodiaquine (MDAQ), lumefantrine (LMF), dihydroartemisinin (DHA), quinine (QN), mefloquine (MQ), artesunate (AS), pyronardine (PND), piperaquine (PPQ) and Proveblue (PVB)**

**Drug**	**Isolate IC**_**50**_				**Resistance or reduced susceptibility**	
	**Mean**	**CI 95%**	**Min**	**Max**	**Cut-off**	**%**
CQ	52.2 nM	29.1-93.9	6.1	346.4	77 nM	50.0
MDAQ	9.8 nM	5.5-17.4	1.9	117.1	61 nM	5.6
LMF	4.1 nM	2.1-8.1	0.61	82.9	115 nM	0
DHA	0.72 nM	0.35-1.50	0.1	5.08	12 nM	0
AS	1.35 nM	0.53-3.42	0.12	5.53	12 nM	0
QN	63.1 nM	30.9-128.7	6.2	1430	611 nM	5.6
MQ	30.1 nM	19.4-46.6	7.1	63.4	30 nM	55.6
PND	5.8 nM	3.3-10.1	0.4	19.7	ND	ND
PPQ	32.2 nM	16.5-62.8	2.5	168.0	ND	ND
PVB	5.3 nM	2.8-10.1	0.9	40.2	ND	ND

Mean: geometric mean.

CI 95%: 95% confidence interval.

ND: not determined.

## Discussion

This report describes the evaluation of the *ex vivo* susceptibility of *P. falciparum* isolates, taken from patients in Dakar, to ten standard or potential anti-malarial drugs. The patients, recruited at the Hôpital Principal de Dakar from November 2013 to January 2014, said that they did not leave Dakar and its surrounding suburbs during the month preceding their malaria attack.

One limitation of this study was the low number of recruited patients (24) during those two months, due to the diminution of malaria prevalence in Senegal. The malaria prevalence in public health facilities decreased from 17.9% in 2007 to 2.6% in 2008 in Dakar [11]. In Dielmo, a village located at 280 km southeast of Dakar and approximately 15 km north of The Gambia border, the prevalence of malaria decreased from 87.2 to 0.3% in children and 58.3 to 0.3% from 1990 to 2012 [12].

The prevalence of isolates with reduced susceptibility to MQ remained high (55.6%) in Dakar, but was relatively stable compared with the previous year (55 to 62%) [3,10]. The level of *in vitro* MQ resistance increased since previous studies conducted in Dakar. In Dakar, the per cent of isolates with decreased susceptibility was 17% in 2001 [13] and 13% in 2002 [7]. MQ prophylaxis failure has been previously described in Senegal [14], and MQ is one of the three anti-malarial drugs recommended for travellers as an anti-malarial prophylaxis in Senegal. Clinical trials are in progress to evaluate the efficacy of MQ for intermittent preventive treatment of infants and pregnant women, whereas MQ is still used for the treatment of uncomplicated malaria in infants in Dakar. Nevertheless, MQ has been employed relatively infrequently in Africa compared to Asia. The combination of artesunate-mefloquine, which is administered to patients in Asia, is not yet used in Senegal. However, scientific data are not available for MQ monotherapy, and very little data are available on the *in vitro* decreased susceptibility to MQ and its clinical implications in Africa. It is important to monitor the evolution of *P. falciparum* susceptibility to MQ, to archive suspicious isolates and to correlate clinical outcomes with pharmacokinetic and phenotypic responses and with molecular markers.

As far back as 1988, *in vitro P. falciparum* resistance to CQ was reported in Dakar, and reports of resistance in other regions of the country followed shortly thereafter [15]. From 1991 to 1995, parasitological failures were observed in 21% of patients in Pikine [16]. The prevalence of *in vitro* CQ resistance then decreased in from 52% in 2002 [7] to approximately 20-25% in Dakar in 2009–2011 [3,10]. In 2013–2014, the prevalence of *in vitro* resistance to CQ in Dakar increased again to 50%. A limitation of these results is the very small number of studied samples. However, this phenomenon was already described in Thies and Pikine. Parasites also became less susceptible to CQ from 2008 (median IC_50_ = 30.7 nM) to 2011 (median IC_50_ = 76.1 nM) in Thies [4]. In Pikine, after a decrease of the prevalence of the *pfcrt* 76 T mutation, involved in CQ resistance, from 64-79% before CQ withdrawal (2000 to 2003) [17-19] to 47-60% [20,21] when amodiaquine plus pyrimethamine-sulphadoxine was the first-line treatment (2004–2005), this prevalence has increased slightly to 59% since ACT has been implemented (2006 to 2009) [19]. It is important to monitor the evolution of *P. falciparum* susceptibility to CQ.

The decrease in CQ resistance parallels the withdrawal of CQ treatment and the introduction of ACT in 2002 in Senegal. However, in 2006, CQ was still being administered to patients. In Dakar in 2006, CQ represented 5.1% of the anti-malarial drugs used in children [22] and 3.5% in 2009 [23]. The rapid dissemination of CQ resistance in Dielmo, despite strictly controlled anti-malarial drug use, argues against the re-introduction of CQ, at least in monotherapy, in places where the resistance allele has dropped to very low levels following the discontinuation of CQ treatment [24]. Despite the re-aquisition of CQ susceptibility, any re-introduction would likely result in a rapid re-emergence of resistant strains. Additionally, the increase of CQ resistance in the hypothetical absence of CQ pressure leads to an avoidance of re-introducing CQ in Senegal. There is an urgent need to evaluate the presence and use of CQ in Dakar and to evaluate the capacity of drug pressure on CQ resistance of the different partners combined with artemisinin derivatives in ACT.

The prevalence of isolates with *in vitro*-reduced susceptibility to MDAQ remains low and stable in 2013 (5.6 *versus* 6% in 2009 and 11.8% in 2010) [3,10]. The resistance to amodiaquine has remained low even after the introduction of artesunate-amodiaquine in 2006 in Senegal. A study in Dakar and Mlomp from 1996 to 1998 demonstrated that monotherapy with amodiaquine remained effective for treating uncomplicated malaria in areas where CQ resistance was prevalent [25]. In 2011–2012, the efficacy of ASAQ was evaluated at 99.3% [26]. However, ACT efficacy and resistance must be monitored because clinical failures, or at least extended parasite clearance times, have been described in Southeast Asia [27-30]. In this context, it is important to implement *in vitro* and *in vivo* surveillance programmes.

No isolate exhibited reduced *in vitro* susceptibility to DHA or to AS. This result is consistent with previous studies that did not show any parasites resistant to AS [7,31,32]. However, high IC_50_ values can be found for artemisinin, with an IC_50_ > 30 nM in Dakar [33] and AS with an IC_50_ > 45 nM [13]. The median IC_50_ values increased from 2008 to 2011 (3.2 to 10.1 nM) with a highest IC_50_ value of 73.1 nM in Thies [4]. In the present study, IC_50_ ranged from 0.1 to 5.08 nM for DHA and 0.12 to 8.53 nM for AS. However, the standard *in vitro* test was not adapted to follow resistance to artemisinin derivatives. The clinical resistance to artemisinin correlated with *in vitro* resistance, manifested by an increase in the ring-stage survival rate after contact with artemisinin (ring survival test) [34,35]. In addition, mutations in the *P. falciparum* K13 gene (PF3D7_1343700) that encodes the kelch propeller domain were associated with *in vitro* resistance to artemisinin and with delayed clearance after artemisinin treatment in southern Asia [30,36,37]. It will be a priority to introduce this new *in vitro* test in Senegal.

The other ACT first-line treatment for uncomplicated *P. falciparum* malaria in Senegal is the combination of artemether-lumefantrine. No isolate presented reduced susceptibility to LMF, and this prevalence was remains under 3% in Dakar since the introduction of ACT [3,10]. In 2011–2012, the efficacy of artemether-lumefantrine was evaluated at 100% in Senegal [26].

A new ACT second-line treatment for uncomplicated *P. falciparum* malaria in Senegal is the combination of dihydroartemisinin-piperaquine (DP). DP (Artekin®, Duo-Cotecxin®, Eurartesim®) is administered as single daily dose for three days. It has been demonstrated to be well tolerated and highly effective for the treatment of uncomplicated *P. falciparum* malaria in Africa [38-40]. In 2011–2012, the efficacy of DP was evaluated at 100% in Senegal [26]. The PPQ IC_50_ values (geometric mean IC_50_ = 32.2 nM) observed in Dakar in 2013 were slightly lower than those found in other *ex vivo* studies in Africa (geometric mean IC_50_ = 81.3 nM and 66.8 nM) [41,42].

The pyronaridine-artesunate combination (Pyramax®) is one of the most recent ACT combinations and is currently under development by the not-for-profit organisation Medicines for Malaria Venture (Geneva, Switzerland) and the pharmaceutical company Shin Poong Pharmaceuticals (Seoul, Republic of Korea) for the treatment of uncomplicated *P. falciparum* malaria and for the blood stages of *Plasmodium vivax* malaria. The efficacy of PND-artesunate was not inferior to that of artemether-lumefantrine in the treatment of uncomplicated *P. falciparum* malaria in Africa [43]. The PND IC_50_ values (geometric mean IC_50_ = 5.8 nM) observed in Dakar in 2013 were comparable to those obtained in Dielmo in 1996 and 1997 (3.8 nM and 4.52 nM) [44,45].

In 2013, 7% of isolates showed low reduced susceptibility to QN, which is in accordance with previous studies in Dakar [3,7,10,13]. Even in areas where QN efficacy remains good, such as sub-Saharan Africa, the susceptibility of individual *P. falciparum* isolates to QN has varied widely. The IC_50_s for isolates collected in Dakar were 6 to 1,291 nM in 2009 [10] and 5 to 1,195 nM in 2010 [3]. The wide range in QN susceptibility and recent evidence for QN treatment failure observed across Africa [46,47] or in Senegal in a patient who spent two months in Dielmo in 2007 [48] suggest that the evolution of parasites with reduced susceptibility may contribute to QN decreased efficacy. However, the 24 patients in this study were successfully treated with QN.

Proveblue (PVB), which is a methylene blue preparation that complies with the European Pharmacopoeia and contains limited organic impurities and heavy metals of recognized toxicity, has previously been demonstrated to possess *in vitro* anti-malarial activity against 23 *P. falciparum* strains that were resistant to various anti-malarial drugs [49]. PVB exhibited noticeable synergistic effects in combination with MQ and QN and high synergistic effects associated with DHA [50]. Treatment with 1 to 10 mg/kg of weight of PVB for five days significantly reduced or prevented cerebral malaria in mice [51-53]. The IC_50_ for PVB ranged from 0.88 nM to 40.2 nM with a mean of 5.3 nM. These data show that PVB is active *in vitro*, in line with previous studies with methylene blue with organic as well as inorganic impurities in parasites from Nigeria, Kenya and Thailande [54-56]. Another advantage of the use of PVB is that methylene blue has gametocytocidal properties and can reduce the transmission of *P. falciparum* [57,58].

Limitations must be taken into account such as the very small number of samples which are not representative of susceptibility in Senegal but only from a facility in Dakar, the Hôpital Principal de Dakar, which certainly selects most severe malaria than in neighborhood clinics.

The introduction of ACT in 2002 in Senegal did not induce a decrease in *P. falciparum* susceptibility to individual drug components, such as DHA, AS, MDAQ, and LMF. The prevalence of *P. falciparum* isolates with reduced drug susceptibility to MQ remains high and stable in Dakar. Since 2004, the prevalence of CQ resistance has decreased, but then rebounded in 2013 in Dakar. PND, PPQ and PVB showed high *in vitro* activity against *P. falciparum* parasites from Dakar. Maximizing the efficacy and longevity of ACT as a tool to control malaria will critically depend on pursuing intensive research into identifying *in vitro* markers as well as implementing *ex vivo* and *in vivo* surveillance programmes.
